# Combining string and phonetic similarity matching to identify misspelt names of drugs in medical records written in Portuguese

**DOI:** 10.1186/s13326-019-0216-2

**Published:** 2019-11-12

**Authors:** Hegler Tissot, Richard Dobson

**Affiliations:** 1 Institute of Health Informatics, University College London, London, UK; 2 Health Data Research UK London, University College London, London, UK; 3 Department of Biostatistics and Health Informatics, Institute of Psychiatry, Psychology and Neuroscience, King’s College London, London, UK

**Keywords:** Phonetic similarity, Similarity search, Misspelt names of drugs

## Abstract

**Background:**

There is an increasing amount of unstructured medical data that can be analysed for different purposes. However, information extraction from free text data may be particularly inefficient in the presence of spelling errors. Existing approaches use string similarity methods to search for valid words within a text, coupled with a supporting dictionary. However, they are not rich enough to encode both typing and phonetic misspellings.

**Results:**

Experimental results showed a joint string and language-dependent phonetic similarity is more accurate than traditional string distance metrics when identifying misspelt names of drugs in a set of medical records written in Portuguese.

**Conclusion:**

We present a hybrid approach to efficiently perform similarity match that overcomes the loss of information inherit from using either exact match search or string based similarity search methods.

## Background

There is a large amount of unstructured data being produced by different kinds of information systems, in a variety of formats, due to the advancement of communication and information technologies [1, 2]. Within the clinical domain, Electronic Health Record (EHR) systems are becoming widely adopted, from which information describing the patient’s health conditions is often presented and stored in the form of free text notes [3]. Existing text-mining methods aim to extract detailed structured information from clinical narratives, such as drug prescriptions, their variability, and adverse drug reactions [4, 5]. However, free-text is susceptible to typing and phonetic misspellings. Spelling errors of generic drug names can occur in up to one out of six entries in electronic drug information systems. Such errors are likely to be responsible for up to 12% of adverse drug events, mainly caused by errors during transcription of prescriptions, illegible prescriptions, or drug name confusion [6]. Due to such frequency and the relevance of drug information in clinical tasks, spelling correction becomes crucial to support health care professionals with spelling error-tolerant engine systems.

Similarity comparison algorithms can be used to identify and extract concepts from free text [7] when text is loaded with misspellings. String similarity metrics (e.g. Edit Distance [8] and Jaro-Winkler Distance [9]) can measure similarity between two strings. These functions can be used to compare the elements from the input data source against an existing dictionary in order to identify a possible valid word matching a misspelling. However, existing string similarity algorithms may be inefficient to analyse text loaded with spelling errors because they may not necessarily handle specific aspects, such as phonetic errors [10]. In these cases, it is necessary to use phonetic similarity metrics.

In order to overcome the possible loss of information by using exact match search methods to find mentions of drugs within patient records, we propose a hybrid solution coupling string and phonetic similarity metrics to identify misspelt names of drugs. This approach was used to produce a dictionary of misspelt variations. A Trie-based fast similarity search algorithm was then able to identify a broader range of potential candidates as misspelt variations for each drug name.

## Use-case

Since July 2013 the Brazilian government tries to address the shortage of doctors, especially in the inner cities and the outskirts of large cities in Brazil, through the hiring of doctors from other countries. With the addition of doctors with distinct language background in the public health system (especially from South and Central America where people originally speak Spanish), a larger number of spelling errors have been found in electronic record systems. Such errors occur mainly due to the similarity of the Portuguese language with other Latin languages (such as Spanish and Italian) [11].

*InfoSaude* (InfoHealth) [12] is an information system created to manage and track medical records, such as exams, vaccinations, and drug prescriptions. The system is used to meet the needs of 75 public health centres in the city of Florianopolis/Brazil. It integrates different information structures used by the Brazilian Ministry of Health, such as the Outpatient Information System (CIS) and the International Code of Diseases (ICD). The system also generates information for Ambulatory Care Individual Report (RAAI), summarizing data on the type of care, pregnancies, procedures performed on the patient, applied vaccines and drug prescriptions. Whilst maintaining a series of structured information, the system also contains textual fields that are filled by health professionals during patient care.

Although *InfoSaude* has structured information about drug prescriptions, a deeper analysis on drug usage, abuse, or checking whether patients are correctly and effectively making use of the prescribed drugs, relies on the observations registered by the clinicians using free text. However, the textual content of the medical records does not go through any kind of review. Thus, it is common to find a number of spelling and phonetic errors that could harm any further analysis. An information extraction system is expected to overcome this problem in order to avoid information loss.

## Approximate string match

The existing similarity match methods range from using basic string similarity distance metrics, which measure inverse similarity between two text strings by providing an algorithm-specific numerical indication of distance, to the use of more sophisticated methods coupled with the phonetic representation of words in a given language.

Edit Distance (ED) (or Levenshtein Distance) [8] is the most widely known string metric. ED operates between two input strings – *ED*(*w*_1_,*w*_2_) – and returns the minimum number of operations (single-character edits) required to transform string *w*_1_ into *w*_2_. Other examples and variations of string similarity metrics include Jaro-Winkler Distance [9], Hamming Distance [13], and *String*_*Sim*_ [14]. However, string distance measures tend to ignore the relative likelihood errors.

Phonetic representations encode words based on the sound of each letter to translate a string into a canonical form. Soundex [15] is an example of a phonetic matching scheme initially designed for English that uses codes based on the sound of each letter to translate a string into a canonical form of at most four characters, preserving the first letter. In addition, phonetic similarity metrics are able to assign a high score even though comparing dissimilar pairs of strings that produce similar sounds [14, 16]. As the result, phonetically similar entries will have the same (or similar) keys and they can be indexed for efficient search using some hashing method. However, phonetics is language-dependent [17, 18] and solutions for this sort of problems must be specially designed for each specific language.

In addition, fast similarity search approaches have been proposed in order to match free text against large dictionaries or databases, being supported by either indexed database structures [14, 19, 20] or Trie-based (prefix index) approximate matching [21–23]. In an initial experiment, Fuzzy Keyword Search [22] has proved to be efficient by combining Trie-based search with string similarity functions. However, processing time grows exponentially as long as the Edit Distance threshold increases, becoming inefficient for *ED*>2, which we were able to confirm by comparing the processing time (in milliseconds) spent to perform 1000 searches over a dictionary of 80,000 entries, varying ED amongst 0 (16 ms), 1 (218 ms) and 2 (3267 ms).

## Method

As part of a NLP pipeline that aims to identify different aspects of drug usage by patients, one of the atomic steps within this pipeline is the identification of drug names in free text. In this section we describe how string and phonetic similarity metrics can be combined to improve accuracy on identifying misspelt names of drugs within a set of records written in Portuguese. Our approach has two main steps. First, we combine string (*String*_*Sim*_) and language-dependent phonetic (*PhoneticMapSim*_*PT*_) similarity metrics proposed in [18] in a hybrid similarity search solution in order to produce a base dictionary of misspelt variations. These metrics were originally designed for the Brazilian Portuguese language. Finally, this dictionary is used as input for a fast Trie-based similarity search algorithm that finds potential candidates to be annotated as drug names in text.

We started using list of 5535 drug names available in the *InfoSaude* system, and searching the most cited drugs in a experimental dataset of clinical notes provided by the *InfoSaude* team (de-identified data with no ethical approval required) from 4748 distinct patients (multiple documents per patient). An exact match search produced a list of 516 drug names, from which the 20 most cited drugs in the text were initially selected (Table 1).

**Table 1:** Occurrence (#) of the 20 most cited drug names in a set of 4748 medical records written in Portuguese

Drug name	Number of occurrences
Fluoxetina	18624
Paracetamol	8697
Diazepam	8474
Amitriptilina	8463
Omeprazol	7825
Dipirona	7320
Glicose	5721
Captopril	5383
Insulina	5290
Nimesulida	4228
Clorpromazina	4226
Enalapril	4144
Imipramina	4135
Sinvastatina	3862
Carbamazepina	3853
Amoxicilina	3716
Ibuprofeno	3714
Metformina	3467
Risperidona	3464
Atenolol	3224

**Fig. 1:**
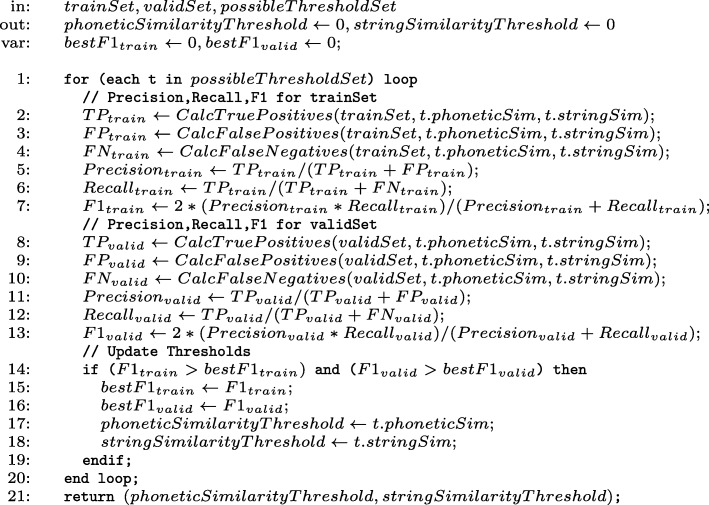
A pseudo-code to find similarity thresholds

In this first step, we aim to produce a base dictionary of misspelt drug name variations by combining string and phonetic thresholds in order to maximise the accuracy on identifying true positive misspelt words. Such thresholds are used to determine whether a candidate misspelt word correspond to a drug name. Inappropriate low threshold values may return too many false candidates favouring low precision by including words with low similarity values that do not correspond to a drug name. In contrast, high threshold values may exclude possible valid misspelt drug names from the final matching, favouring low recall. The method used to find the most suitable string and phonetic similarity thresholds is described below:

- We selected a list of candidate words (similar words) for each drug, by finding all words that have at least 3 matching consonantal phonemes in each pair of true positive and candidate drug name or the Edit Distance metric ≤3.
- The returned list of similar words corresponding to a given drug name *d* was manually analysed. We applied a filter in order to consider candidates words *w* where *String*_*Sim*_(*d, w*)≤0.6 (this threshold can be considered relatively low and resulted approximately 50% of false positive candidates). The final result is a list of 1791 distinct candidate words for the set of drug names listed in Table 1 – an average of 90 similar candidate words per drug.
- The candidate words were manually annotated to identify whether each word corresponds to a valid drug name, resulting 938 positive matches and 853 negative matches. We also automatically annotated each positive and negative match with the corresponding string and phonetic similarity measures (StringSim and PhoneticMapSimPT).
- We used the annotated set of candidates to perform a grid search over the combined string and phonetic similarity values in order to find the best similarity threshold values that favour precision and recall. The list of 20 drugs was split into two groups (10 drugs each) used as training and validation sets. The grid search algorithm is presented in the form of a pseudo-code in Fig. 1.

The pseudo-code performs an exhaustive search for the best pair of phonetic and string similarity thresholds. The input comprises two manually annotated lists (trainSet and validSet) – containing names of drugs and candidate similar words with the corresponding positive or negative match flag – and a list with 7730 pairs of possible string and phonetic threshold values. 660 pairs of similarity values contain StringSim = 0, i.e. a possible solution considering only the phonetic similarity metric as a threshold. Finally, for each possible pair of threshold values, the algorithm calculates Precision, Recall, and F1 for each set of 10 drugs (trainSet – lines 2-7 – and validSet – lines 8-13). The final thresholds are updated each time both *F*1_*train*_ and *F*1_*valid*_ simultaneously achieve better values – lines 14-19. After executing the described pseudo-code on the data extracted from the medical record set, we observed a hybrid solution considering both phonetic and similarity thresholds achieved better accuracy on identifying misspelt names of drugs. The hybrid solution combines a smaller phonetic threshold to perform a fast similarity search that result more similar words, coupled with a string similarity threshold that works as a complementary filter. Table 2 depicts the final resulting threshold values.

**Table 2:** Best threshold values found by the grid search method

	Parameter	Value
Training Set	Number of true positives	417
	Number of false positives	31
	Number of false negatives	25
	Precision	0.931
	Recall	0.943
	F1-score	0.937
Validation Set	Number of true positives	477
	Number of false positives	39
	Number of false negatives	19
	Precision	0.924
	Recall	0.961
	F1-score	0.942
Thresholds	Phonetic similarity	0.844
	String similarity	0.831

## Results

The final threshold values were used to find positive misspelt names for a broader list of drugs. A total of 1442 misspelt words corresponding to 409 different drug names were identified. Table 3 shows the drug names (except those occurring in the training and validation sets) in which the greatest number of misspelt forms were found, as well as the corresponding accuracy (precision, recall, F1) on identifying the misspelt variations for each drug. We also compare the accuracy of our approach against the widely used Edit Distance metric.

**Table 3:** Drugs with the highest number of misspelt variations

Drug name	Number of similar words	Inexact Phonetic Match			F1-Score when using only string match			
		Precision	Recall	F1	*ED*≤1	*ED*≤2	*ED*≤3	*ED*≤4
Propanolol	52	0.960	0.979	**0.967**	0.310	0.819	0.945	0.955
Glibenclamida	49	1.000	1.000	**1.000**	0.829	0.956	0.955	0.961
Anlodipino	49	0.913	0.976	0.944	0.612	0.938	**0.952**	0.942
Medroxiprogesterona	47	1.000	0.914	**0.955**	0.763	0.881	0.955	0.927
Metoclopramida	46	1.000	0.977	**0.989**	0.750	0.977	0.965	0.964
Loratadina	46	0.837	0.947	0.889	0.774	**0.973**	0.963	0.955
Dexametasona	45	1.000	0.800	0.889	0.615	0.915	**0.954**	0.952
Furosemida	43	0.963	1.000	0.981	0.844	**1.000**	0.976	0.961
Prednisona	42	1.000	0.878	0.935	0.730	0.952	**0.976**	0.956
Hidroclorotiazida	41	1.000	0.975	**0.987**	0.776	0.962	0.952	0.940
Diclofenaco	41	0.923	0.947	0.935	0.812	0.914	**0.950**	0.912
Ciprofloxacino	37	1.000	0.918	**0.958**	0.520	0.878	0.935	0.922
Espironolactona	36	1.000	1.000	**1.000**	0.714	0.941	0.948	0.962
Salbutamol	36	1.000	0.972	**0.986**	0.819	0.956	0.976	0.943
Clonazepam	34	1.000	1.000	**1.000**	0.692	0.969	0.961	0.939
Beclometasona	33	1.000	0.967	**0.984**	0.777	0.935	0.961	0.921
Dexclorfeniramina	31	1.000	0.903	0.949	0.708	0.872	**0.960**	0.959
Metronidazol	30	0.965	0.965	**0.965**	0.816	0.964	0.942	0.926
Prednisolona	30	0.965	0.965	0.965	0.739	0.925	**0.976**	0.966
Isossorbida	29	0.963	1.000	**0.981**	0.761	0.960	0.957	0.936
Average F1-score				**0.963**	0.718	0.934	0.958	0.945

The best F1 score is highlighted for each drug

Information Extraction and NLP systems are traditionally evaluated through precision, recall, and F1-score relevance measures. Precision is equivalent to the amount of retrieved instances that are relevant, while recall is equivalent to the amount of relevant instances that are retrieved. The terms *true positives* (TP) and *true negatives* (TN) represent the correct result and the correct absence of results respectively, while the terms *false positives* (FP) and *false negatives* (FN) correspond to the unexpected result and the missing result respectively. These terms are used to define precision and recall according to Eqs. 1 and 2. In other words, the greater is precision the lesser is the proportion of false positive results, whilst the greater is recall the lesser is the proportion of false negative results. Finally, the F1-score result can be interpreted as the weighted average (or harmonic mean) between precision and recall [24], reaching its best value at 1 and worst score at 0 (Eq. 3).

1 $$ Precision = \frac{TP}{TP + FP}  $$

2 $$ Recall = \frac{TP}{TP + FN}  $$

3 $$ F1 = 2 \times \frac{Precision \times Recall}{Precision + Recall}  $$

Some drugs reach recall lower than 0.9 (e.g. “*Dexametasona*” and “*Prednisona*”) and “*Loratadina*” has a precision around 0.83, which is lower than most others. Although not being conclusive and still needs further investigation, we found in an initial analysis the observed differences among the scores refer to some prefixes (e.g. “*cap*”, “*clo*”, “*para*”, “*ox*”) and suffixes (e.g. “*mina*”, “*lina*”, “*pina*”, “*tina*”) that are used to compound names of distinct drugs, increasing the value of the similarity scores for negative matches, thus leading to false positives. Some words in Portuguese can also be compound by the verbal derivative form of a noun, such as “*insulinizar*” as a verb referring to the substance “*Insulina*”. All these factors combined increase the probability of a drug name being similar to a more diverse set of distinct words or other drugs in this specific language.

A hybrid solution showed to be efficient on dealing with both phonetic and spelling errors, and combining both string and phonetic similarity thresholds favoured precision and recall when looking for misspelt drug names. However, this approach suffers in terms of performance in a large corpus. Thus, we used the resulting dictionary of true positive misspelt names of drugs as input for an adapted version of the Trie-based fast search approach algorithm proposed in [22]. This combined approach showed to be efficient (in terms of performance) on finding dictionary-based variations with *max*(*ED*(*word*_1_;*word*_2_))=1. As a result, hundreds of potential misspelt variations for drug names were identified after processing a new set of medical records comprising approximately 5 million documents. To illustrate the potential use of such combined method, 231 positive misspelt variations for “*Fluoxetina*” (Fluoxetine) and 501 positive misspelt variations for “*Paracetamol*” have been already positively identified. Table 4 shows that some of this variations for the drug “*Fluoxetina*” can have high values for the Edit Distance metric.

**Table 4:** Examples of misspelt variations for “*Fluoxetina*” (Fluoxetine) and the corresponding Edit Distance (ED) values

Misspelt variation	ED
dfluoxetina	1
flluoxetina	1
floxetina	1
fluoexetina	1
fluoixetina	1
fluopxetina	1
fluoxertina	1
fluoxetiina	1
fluoxetijna	1
fluoxetin	1
fluoxetinas	1
fluoxetna	1
fluoxetona	1
fluoxettina	1
fluoxetuina	1
fluoxewtina	1
fluoxtina	1
fluozxetina	1
fluuoxetina	1
fluuoxetina	1
fluxetina	1
fluyoxetina	1
flhuoxetin	2
flluoxetin	2
flouxetina	2
fluoxeitna	2
fluoxetian	2
fluxoetina	2
fluxotina	2
fluloextina	3
fluoxetinaate	3
fluoxetinapor	3
flxtina	3
fluoxetinapara	4
infloexetina	4

## Conclusions and future work

In this paper, we presented a hybrid similarity approach that efficiently performs a joint string and language-dependent phonetic similarity search over a set of medical records written in Portuguese. Experimental results showed this method is potentially accurate and able to identify misspelt names of drugs, overcoming the loss of information inherit from using either exact match search methods or string based similarity search. We coupled the proposed approach with a Trie-based fast similarity search algorithm that is able to use small Edit Distance threshold (≤1) over the produced dictionary of misspelt names in order to find a broader number of misspelt variations within an affordable processing time in a large corpus.

Some of the directions in which this work can be extended include: a) adapting the phonetic matching process originally designed to the Portuguese language to be used over large corpora in different languages, such as English; b) integrating our method in a framework for Medical Records Information Extraction applications to address the problem of generically dealing with spelling errors in the information extraction process beyond names of drugs, including other types of clinical variables, such as symptoms and diagnoses; c) exploring the use of machine learning methods to optimally and dynamically tune the threshold parameters and disambiguating misspelt candidates in cases when they are similar to more than one medication; d) comparing the proposed solution with other approximate string match approaches.

## Data Availability

The dataset is available at http://github.com/HeglerTissot/mnd, including the complete set of drug names and words used in our model, as well as the pre calculated values for the string and phonetic similarity matching metrics for each pair (*d**r**u**g, w**o**r**d*).

## Abbreviations

CIS: Outpatient Information System
ED: Edit Distance (or Levenshtein Distance)
EHR: Electronic Health Record
HealTAC: Healthcare Text Analytics Conference
ICD: International Code of Diseases
NIHR: National Institute for Health Research
RAAI: Ambulatory Care Individual Report
